# Neuroprotective Mechanisms of Glucagon-Like Peptide-1-Based Therapies in Ischemic Stroke: An Update Based on Preclinical Research

**DOI:** 10.3389/fneur.2022.844697

**Published:** 2022-03-15

**Authors:** Xiaoyan Yang, Qiang Qiang, Nan Li, Peng Feng, Wenshi Wei, Christian Hölscher

**Affiliations:** ^1^Department of Neurology, Huadong Hospital Affiliated to Fudan University, Shanghai, China; ^2^Department of Neurology, The Second Affiliated Hospital of Shanxi Medical University, Taiyuan, China; ^3^Henan University of Chinese Medicine, Academy of Chinese Medical Science, Zhengzhou, China

**Keywords:** stroke, GLP-1, GLP-1R agonists, neuroprotection, diabetes

## Abstract

The public and social health burdens of ischemic stroke have been increasing worldwide. Hyperglycemia leads to a greater risk of stroke. This increased risk is commonly seen among patients with diabetes and is in connection with worsened clinical conditions and higher mortality in patients with acute ischemic stroke (AIS). Therapy for stroke focuses mainly on restoring cerebral blood flow (CBF) and ameliorating neurological impairment caused by stroke. Although choices of stroke treatment remain limited, much advance have been achieved in assisting patients in recovering from ischemic stroke, along with progress of recanalization therapy through pharmacological and mechanical thrombolysis. However, it is still necessary to develop neuroprotective therapies for AIS to protect the brain against injury before and during reperfusion, prolong the time window for intervention, and consequently improve neurological prognosis. Glucagon-like peptide-1 receptor agonists (GLP-1 RAs) are broadly regarded as effective drugs in the treatment of type 2 diabetes mellitus (T2DM). Preclinical data on GLP-1 and GLP-1 RAs have displayed an impressive neuroprotective efficacy in stroke, Parkinson's disease (PD), Alzheimer's disease (AD), Amyotrophic lateral sclerosis (ALS), and other neurodegenerative diseases. Based on the preclinical studies in the past decade, we review recent progress in the biological roles of GLP-1 and GLP-1 RAs in ischemic stroke. Emphasis will be placed on their neuroprotective effects in experimental models of cerebral ischemia stroke at cellular and molecular levels.

## Highlights

- Glucagon-like peptide 1 (GLP-1) is a target for the treatment of diabetes mellitus.
- GLP-1 and GLP-1 receptor agonists (GLP-1 RAs) have protective effects in stroke models.
- Many studies have revealed that GLP-1 and GLP-1 RAs can reduce infarct volume, improve neurological symptoms and prognosis.
- These neuroprotective effects may be mediated by GLP-1 receptors or other pathways.
- GLP-1 RAs are very potential for stroke and more studies are needed to elucidate the mechanisms underlying neuroprotection.

## Introduction

Stroke is a main reason of mortality and disability, there are 80.1 million prevalent cases of stroke around the world (1), resulting in an annual economic burden worldwide. Stroke is clinically defined as a neurological deficit attributed to an acute focal injury of the central nervous system (CNS) (i.e., brain, spinal cord, or retina) by a vascular cause (i.e., infarction, hemorrhage) (2). The burden of disability due to stroke is highest in Asia, the stroke belt of the United States and low- and middle-income countries (3). Acute ischemic stroke (AIS) is known to account for 84.4% of prevalent strokes (4). Results from the Global Burden of Diseases, Injuries, and Risk Factors Study 2015 (GBD 2015) demonstrate that although the prevalence of stroke and age-standardized death rate have decreased as time goes by, there has recently been a significant increase in the numbers of people dying from and affected by stroke, resulting in greater loss of health over the lifespan (5).

For a quarter century, thrombolysis has become a standard of care treatment for AIS (6). Tissue plasminogen activator (tPA) is one of the most biologically effective thrombolytic agents for AIS (7). Nevertheless, this treatment benefits <10% of patients suffered AIS, because tPA must be given within 4.5 h of stroke onset (8). For about 20 years, intravenous tissue plasminogen activator (IV-tPA) remained the dominant treatment until 2015, when more complex clinical trials showed favorable results in endovascular therapy (EVT) (9). Studies have shown that EVT of ischemic stroke with intra-arterial thrombolysis (IAT) and/or the utility of clot-retrieval, stent retriever, and thrombus aspiration devices produced early recanalisation and reperfusion and improved neurological prognosis (10). The therapy of AIS has evolved in the last 5 years with the recognition of the value of endovascular thrombectomy (that is, mechanical clot-retrieval *via* catheter angiography) (11) in properly selected patients, or the utility of brain imaging techniques to individualize the use of thrombectomy up to 24 h after stroke onset including wake-up stroke (WUS) (12, 13). While the value of these major advances is indisputable, actually few patients with ischemic stroke receive endovascular thrombectomy, and fewer than half of those who received treatment show everlasting benefits (14–17). Even with endovascular thrombectomy and/or intravenous thrombolysis, the reduction of disability is highly time-dependent. Controversially, some patients cannot benefit from this therapy because they are treated too late and they have little or no ischemic penumbra to salvage at the time of treatment. Therefore, there is still a great need to develop neuroprotective agents for AIS to protect the brain against injury before and during reperfusion, prolong the time window for interventional treatment as well as further ameliorate functional outcomes. Diabetes is an independent risk factor for stroke, tripling the risk of stroke in diabetics, and stroke accounts for about 20% of deaths in people with diabetes (18). This association suggests that there is a shared mechanism between diabetes and stroke. A combination of medical therapy and behavioral modification has been proven to lower stroke risk in diabetics (19, 20). It is interesting to note that controlling glucose merely does not reduce the risk in diabetics which can be reduced by behavior modification plus medical intervention (21, 22). Recent studies indicate that drugs targeting the glucagon-like peptide-1 receptor (GLP-1R) have neuroprotective effects against stroke.

## Glucagon-Like Peptide-1 and Glucagon-Like Peptide-1 Receptors

Glucagon-like peptide-1 (GLP-1), a product of the cleavage of proglucagon in L cells of intestinal epithelium, is mainly secreted as GLP-1(7-36) NH2, an amidated 30-amino acid peptide (23, 24). It acts by binding to the GLP-1 receptor (GLP-1R) which belongs to the G protein-coupled receptor family. The multiple metabolic effects of the drug GLP-1 include the promotion of insulin secretion in a glucose- dependent way (25, 26), inhibition of appetite and ingestion (27), reduction of gastric emptying (27, 28), stimulation of rodent ß-cell proliferation (29), and increase of natriuretic (30), and diuretic processes (31). Meanwhile, GLP-1 has neuro- and cardioprotective effects, such as inhibiting apoptosis (32) and inflammation (33), and has an impact on memory and learning (34, 35), palatability and reward behavior (36). The beneficial effects of GLP-1 on the CNS are mainly shown in rodent models of stroke, PD, AD, and ALS (37–39). However, it has been claimed that GLP-1 still acts on some extrapancreatic tissues in the absence of the GLP-1R, implying that the hormone can exert effects through presently unidentified receptors or mechanisms as well.

Unlike many gastrointestinal peptides previously found to be insulinotropic in healthy humans or *in vitro*, but without effect in diabetes (24, 40), GLP-1 has a potent antidiabetic effect. Natural GLP-1 has a very short half-life of around 1–2 min (41–43), depending on the species and results from the effects of the enzyme dipeptidylpeptidase-4 (DPP-4) and renal elimination. With enhanced potency and persistence through biochemical modification, GLP-1 receptor agonists (GLP-1 RAs) have become effective agents for treating T2DM and are widely used worldwide. At present, the GLP-1 receptor agonists exendin-4 (Exenatide, Byetta^®^, Bydureon^®^) (44, 45), liraglutide (Victoza^®^) (46), albiglutide [Eperzan^®^ (EU) Tanzeum^®^ (US)] (47, 48), dulaglutide (Trulicity™^®^) (49), lixisenatide (Lyxumia^®^, Adlyxin^®^) (50), semaglutide (Ozempic^®^, Rybelsus^®^) (51, 52), are approved to treat T2DM (53) (see Table 1). These analogs are administered orally or subcutaneously and are well-tolerated by patients. In addition, other relevant therapies based on incretins, including GIP receptor (GIP-R) agonists, dual GLP-1R/GIPR agonists, GLP-1R/GIPR/Glucagon receptor (GCGR) triagonists, and oxyntomodulin (OXM), have been shown to improve outcomes of T2DM in a series of preclinical trials (55, 56).

**Table 1:** Characteristics of GLP-1 RAs that have been approved to treat type 2 diabetes as of 2021, modified from Li et al. (54).

**Generic name**	**Proprietary name**	**Half-life**	**Frequency/administration**	**Company**	**Approved by**
Exenatide	Byetta®	2.4 h	Twice daily/s.c.	Amylin-Astra Zeneca	U.S.FDA, 2005; EMA, 2006
Exenatide LAR	Bydureon®	~2 w	Once weekly/s.c.	Amylin-Astra Zeneca	U.S.FDA, 2012
Liraglutide	Victoza®	13 h	Once daily/s.c.	Novo Nordisk	U.S.FDA, 2010; EMA, 2009
Albiglutide	Tanzeum(US)®	5 d	Once weekly/s.c.	Glaxo SmithKline	U.S.FDA, 2014
	Eperzan(EU)®	5 d	Once weekly/s.c.	Glaxo SmithKline	EMA, 2014
Dulaglutide	Trulicity®	5 d	Once weekly/s.c.	Eli Lilly	U.S.FDA, 2014
Lixisenatide	Lyxumia®	3 h	Once daily/s.c.	Sanofi-Aventis	EMA, 2013
	Adlyxin®	3 h	Once daily /s.c.	Sanofi-Aventis	U.S.FDA, 2016
Semaglutide	Ozempic®	5.7–6.7 d	Once weekly/s.c.	Novo Nordisk	U.S.FDA, 2017; EMA, 2019
	Rybelsus®	5.7–6.7 d	Once daily/Oral	Novo Nordisk	U.S.FDA, 2019

*FDA, The Food and Drug Administration; EMA, European Medicines Agency; s.c.,subcutaneous; LAR, long-acting release*.

It has been proven that the native peptide GLP-1, GLP-1 receptor agonists and GIP-1 receptor agonists can cross the blood-brain barrier (BBB) (57–61). There is also a rising interest in dual GLP-1R/GIPR agonists as neuroprotective drugs that act on respective homoreceptors located in the central nervous system (CNS), with proof that these peptides could also pass through the BBB (62–64).

The GLP-1 receptor belongs to the G protein-coupled receptor (GPCR) B family, consisting of seven transmembrane helices (TMH) interconnected by intracellular loops, with a C-terminal intracellular domain and a large (~120 amino acid) N-terminal extracellular domain (ECD) (65). GLP-1 receptors are substantively expressed and are most abundant in the pancreas, gut and the CNS, but also in the peripheral nervous system (PNS), heart, vasculature, kidneys, and lungs (66).

GLP-1 receptor is widely distributed in the CNS, including the striatum, hypothalamus, cortex, subventricular zone, and substantia nigra, as well as in the brain stem (67–69). In the mammalian brain, including humans and rodents, the expression of GLP-1 receptors has been detected in endothelial cells, microglia, astrocytes, and neurons (70–74). Because the GLP-1 receptors are highly conserved across species, the physiological importance of the peptide and its receptor in a wide range of mammal species is emphasized. Under normal physiological conditions, the expression of the GLP-1 receptor is largely confined to large output neurons, clustered in Purkinje cells, pyramidal cells, and dentate granule cells, where it particularly located on dendrites and on or near synapses (69).

Studies have revealed that the expression of GLP-1 receptor is increased in neurons, GABAergic interneurons, microglia, astrocytes, and endothelial cells in the brain following the AIS (68, 75–77). GLP-1 RAs can alleviate brain inflammation by inhibiting the activation and recruitment of glial cells both *in vivo* and *in vitro* following a variety of injury paradigms (38, 78–84). Activation of GLP-1 receptors also results in nonglycemic effects in a variety of tissues, by acting directly on tissues expressing incretin receptors, as well as through indirect mechanisms regulated by endocrine and neuronal pathways.

The most common side effects of the GLP-1 RAs are gastrointestinal (GI)-related adverse events (AE), such as diarrhea, emesis, and nausea (85), which usually occur during the up-titration phase and are dose-dependent.

## GLP-1 and GLP-1 Receptor Agonists for the Treatment of Stroke

Over the past several years, neuroprotective effects of GLP-1 and GLP-1 Ras have been shown in animal models of stroke, and advances in this area have now been updated. In particular, we focus on data showing GLP-1 and the GLP-1 RAs mediated efficacy against stroke.

### Search Strategy and Selection Criteria

We searched the PubMed (https://pubmed.ncbi.nlm.nih.gov) for English language manuscripts. We used the search terms “stroke,” “GLP-1,” “GLP-1 receptor agonist,” “diabetes,” and “neuroprotection” from 1 January 2011 to 1 August 2021. We mainly selected publications from the past 10 years, but also included highly regarded older and frequently cited publications. Moreover, we also searched the reference lists of articles identified by the search strategy and selected those we judged relevant, and Major trials or studies are referenced to support level 1 evidence and review articles are referenced to provide readers with details. A total of 45 papers were included (see Tables 2A–H).

**Table 2A:** A review of preclinical studies of GLP-1 and GLP-1RAs in stroke.

**References**	**Substance**	**Stroke** **model**	**Occlusion** **time (min)**	**Species**	**Timing of administration**		**Comorbidity**	**Main outcomes**
					**Pre-ischemia**	**Post-ischemia**		
Zhao et al. (86)	RhGLP-1 (7–36)	pMCAO	Permanent	Rat	Before reperfusion, i.p.		Diabetic	RhGLP-1: Infarct volume↓, neurological deficit↓, MDA↓, eNOS↑, SOD↑, iNOS↓, GSH-PX↑.
	Nimodipine				Before reperfusion, i.p.			Nimo: Neurological deficits↓, infarct volume↓, MDA↓, SOD↑, GSH-PX↑, iNOS↓, eNOS↑.
Zhang et al. (87)	Pro-GLP-1	tMCAO	90	Mice	1 w, qd, i.p.	–	–	GLP-1↑, neurological deficits↓, infarct volume↓, Bax↓, Bcl-2↑, caspase-3↓.
Jiang et al. (88)	RhGLP-1	tMCAO	90	Rat	2 w, tid, i.p.	–	Diabetic	RhGLP-1: FBG↓, neurological deficits↓, infarct volume↓, S100B↓, NSE↓, MBP↓.
	Nimodipine				2 w, tid, i.p.	–		Nimo: Neurological deficits↓, infarct volume↓, S100B↓, MBP↓.
	Insulin aspart				2 w, tid, i.p.			Ins: FBG↓, neurological deficits↓, infarct volume↓.
Fang et al. (89)	RhGLP-1	tMCAO	120	Rat	–	2 h → 3 d, tid, i.p.	Diabetic	RhGLP-1: FBG↓, Neurological deficits↓, infarct volume↓, Nrf2↑, HO-1↑, p-PI3K/PI3K↑, SOD↑, MDA↑.
	Nimodipine				–	2 h → 3 d, tid, i.p.		Nimo: Neurological deficits↓, infarct volume↓, Nrf2↑, HO-1↑, SOD↑, MDA↑.
	Insulin				–	2 h → 3 d, tid, i.p.		Ins: FBG↓, Nrf2↑, HO-1↑, p-PI3K/PI3K↑, SOD↑, MDA↑.
Fang et al. (90)	RhGLP-1	tMCAO	90	Rat	2 w, tid, i.p.	–	Diabetic	RhGLP-1: Neurological deficits↓, FBG↓, infarct volume↓, MDA↓, GSH↑, SOD↑, EAAT2↑, cleaved caspase-3↓, Bcl-2/Bax↑.
	Nimodipine				2 w, tid, i.p.	–		Nimo: Infarct volume↓, neurological deficits↓, MDA↓, GSH↑, EAAT2↑, SOD↑, cleaved caspase-3↓, Bcl-2/Bax↑.

**Table 2B:** A review of preclinical studies of GLP-1 and GLP-1RAs in stroke.

**References**	**Substance**	**Stroke** **model**	**Occlusion** **time (min)**	**Species**	**Timing of administration**		**Comorbidity**	**Main outcomes**
					**Pre-ischemia**	**Post-ischemia**		
Huang et al. (91)	GLP-1 (9–36)	tMCAO	60	Mice	1 w, qd, i.p.	–	–	Neurological deficits↓, infarct volume↓, NF-κB-p65↓, p- AKT↑.
Lee et al. (77)	Exendin-4	BCCAO	5	Gerbil	2 h, i.p.	1 h, i.p.	–	Neuronal death delay, GLP-1R↑, Iba-1↓, and independent of endothelin receptor.
Teramoto et al. (78)	Exendin-4	tMCAO	60	Mice	–	Onset, 1, 3 h, i.v.	–	Infarct volume↓, neurological deficit↓, inflammatory response (Iba-1↓, iNOS↓), cell death↓, cAMP↑, p-CREB↑, oxidative stress markers (8-OHdG↓, HHE↓).
Briyal et al. (92)	Exendin-4	pMCAO	Permanent	Rat	1 w, bid, i.p.	–	–	Oxidative stress markers (SOD↑,MDA↓, GSH↑), infarct volume↓, neurological deficit↓.
Darsalia et al. (93)	Exendin-4	tMCAO	90	Rat	4 w, bid, i.p.	2/4 w, bid, i.p.	Diabetic	NeuN↑, Iba-1↓, ED1↓, Ki67↑, DCX↑.
Darsalia et al. (94)	Exendin-4	tMCAO	30	Mice	–	1.5/3/4.5 h → 1 w, qd, i.p.	Diabetic/obese	Proinflammatory markers (MCP-1↓, IL-1β↓), NeuN↑, M2 markers (CD206↑, Arg1↑, YM1/2↑).
Jin et al. (95)	Exendin-4	BCCAO	7	Gerbil	30 min, i.p.	30 min → 2 d, bid, i.p.	–	NeuN↑, Fluoro-Jade B↓, Bcl-2/Bax↑, HIF-1α↓.
Jia et al. (76)	Exendin-4/	tMCAO	60	Rat	15 min, i.c.v.	–	–	Infarct volume↓, neurological deficit↓, β-endorphin↑.
		Exendin 9-39			15 and 15 min after first one, i.c.v.		–	Ex 9–39: Prevented neuroprotection of Ex-4.
Chien et al., (96)	Exendin-4/PEx-4	BCCAO	10	Rat	–	24h, s.c.	Diabetic	Ex-4: Cerebral blood flow and microcirculation↑, gp91↓, CHOP↓, GFAP↓, ICAM-1↓, NF-κB↓, cognition deficit↓, p-eNOS↑, TUNEL↓, caspase-3↓, p-Akt↑, PARP↓, Bax/Bcl-2↓, ICAM-1↓. PEx-4 was more effective than Ex-4.

**Table 2C:** A review of preclinical studies of GLP-1 and GLP-1RAs in stroke.

**References**	**Substance**	**Stroke model**	**Occlusion time (min)**	**Species**	**Timing of administration**		**Comorbidity**	**Main outcomes**
					**Pre-ischemia**	**Post-ischemia**		
Zhang et al. (97)	Exendin-4	tMCAO	90	Mice	1 w, q d, i.n./i.p.	–	–	Infarct volume↓, neurological deficit↓, cAMP/PKA/p-CREB↑, PI3K/p-Akt↑, caspase-3↓, neuroprotection of intranasal Ex-4 depended on activation of GLP-1R.
Kuroki (98)	Exendin-4	tMCAO	60	Mice	–	60 min, i.p.	Hyperglycemia	Infarct volume↓, edema volume↓, neurological deficit↓, survival rate↑, MMP-9↓, BBB permeability↓, Iba-1↓, neutrophil infiltration↓, TNF-α↓, oxidative stress markers (DNP↓).
Li et al. (99)	Exendin-4/	tMCAO	60	Mice	–	Onset/3/6/12 h,i.p.	Diabetic	Ex-4: Oxidative stress markers (ROS↓, ICAM-1↓, MDA↓ DHE↓), edema volume↓, cerebral microcirculation↑, apoptosis markers (TUNEL↓, caspase-3↓), MnSOD↓, PARP↓,p eNOS↑, Bax/Bcl-2↓, NF-κB p50 and p65↓, p-Akt↑, voiding impairments↓, cognition deficit↓.
	Liraglutide				–	Onset/3/6/12h,i.p.		Lir: Oxidative stress markers (ROS↓, ICAM-1↓, MDA↓ DHE↓), edema volume↓, cerebral microcirculation↑, apoptosis markers (TUNEL↓, caspase-3↓), MnSOD↓, PARP↓,p eNOS↑, Bax/Bcl-2↓, NF-κB p50 and p65↓, p-Akt↑, voiding impairments↓, cognition deficit↓.
Chen et al. (71)	Exendin-4	tMCAO	45	Mice	–	Onset, i.v.	Hemorrhagic transformation	Infarct volume↓, neurological deficit↓, PI3K/Akt/GSK-3β↓, claudin-3↑, p-β-catenin/β-catenin↓, TNF-α↓, claudin-5↑, ICAM-1↓, IL-1β↓, IKK-β↓, VCAM-1↓, 8-OHdG↓, HHE↓, MPO↓, Iba1+/TNF-α↓, NF-κB↓.

**Table 2D:** A review of preclinical studies of GLP-1 and GLP-1RAs in stroke.

**References**	**Substance**	**Stroke model**	**Occlusion time (min)**	**Species**	**Timing of administration**		**Comorbidity**	**Main outcomes**
					**Pre-ischemia**	**Post-ischemia**		
Yang et al. (100)	Exendin-4	tMCAO	60	Rat	–	1/2/3/4/5/7/10d, qd, i.v.	–	APE1↑, γH2AX↓, PI3K/p-AKT↑/p-CREB↑.
Kim et al. (73)	Exendin-4/	tMCAO	60	Rat	30 min, i.c.v.	–	–	Ex-4: Infarction volume↓, GLP-1R↑, cAMP↑, IB1/JIP1↑, p-SAPK↓/p-JNK↓, COX-2↓, PGE2↓.
	Exendin 9-39				30 min, i.c.v.	–	–	Ex 9-39: infarction volume↓, GLP-1R↓, p-JNK↑.
Shan et al. (101)	Exendin-4/	tMCAO	90	Rat	–	Onset, i.p.	–	Ex-4: Neurological deficit↓, infarct volume↓, MCP-1↓, MMP-9↓, IL-1β↓, CXCL-1↓, VEGF-A↓, IL-6↓, ZO-1↑, PLCγ↓/PKCα↓/eNOS↓, p-JAK2↓/p-STAT3↓.
	Exendin 9-39				–	Onset, i.p.	–	Ex 9-39: neuroprotection of Ex-4 was blocked by combination with Ex 9-39.
Zhang et al. (102)	Exendin-4	tMCAO	60	Mice	1/3/7/14 d, qd, i.p.	–	–	Infarct volume↓, neurological deficit↓, p-PI3K↑/p-AKT↑, p-mTOR↑, HIF-1α↑.
Augestad et al. (103)	Exendin-4	tMCAO	30	Mice	–	3 d → 6/8 w, qd, i.p.	Diabetic/obese	Insulin sensitivity↑, Iba-1↓, CD68↓, vessel density↑, CD13+↑, neurological deficit↓.
Nizari et al. (104)	Exendin-4/	tMCAO	90	Rat	20/10 min before reperfusion; i.v.		RIC	RIC: Infarct volume↓, neurological deficit↓. Ex-4: GLP-1R↑, PO2↑, PtO2↑.
	Exendin 9-39				10 min prior to the first episode of RIC			Ex 9-39: blocked the neuroprotective effect of RIC.
Sato et al. (105)	Liraglutide	tMCAO	90	Rat	–	1 h, i.p.	–	Neurological deficit↓, oxidative stress markers (d-ROMs↓), infarct volume↓, VEGF↑.

**Table 2E:** A review of preclinical studies of GLP-1 and GLP-1RAs in stroke.

**References**	**Substance**	**Stroke model**	**Occlusion time (min)**	**Species**	**Timing of administration**		**Comorbidity**	**Main outcomes**
					**Pre-ischemia**	**Post-ischemia**		
Briyal et al. (106)	Liraglutide	pMCAO	Permanent	Rat	2 w, s.c.	–	Diabetic	Infarct volume↓, oxidative stress markers (MDA↓, GSH↑, SOD↑), apoptosis-related protein (Bcl-2↑, Bax↓), neurological deficit↓.
Zhu et al. (107)	Liraglutide	pMCAO	Permanent	Rat	–	1/2/7 d, qd, s.c.	–	Neurological deficit↓, infarct volume↓, Bcl-xl/Bad↑, p-P38↓, JNK↓, Bcl-2/Bax↑, TUNEL↓, caspase-3↓/−8↓/-9↓, PARP↓, p-ERK↑, ROS↓, p-AKT↑.
Dong et al. (108)	Liraglutide	tMCAO	90	Rat	–	1 d → 4 w, qd, s.c.	–	Neurological deficit↓, glucose metabolism (18F FDG↑), GFAP↓, GLP-1R↑, NeuN↑, vWF↑.
Deng et al. (109)	Liraglutide	pMCAO	Permanent	Rat	1 w, bid, i.p.	1 w, bid, i.p.	Diabetic	Neurological deficit↓, infarct volume↓, HO-1↓, Nrf2↓, oxidative stress (MPO↓, SOD↑).
Chen et al. (110)	Liraglutide	pMCAO	Permanent	Mice	–	1 d → 2 w, qd, i.p.	–	Infarct volume↓, neurological deficit↓, VEGF↑, BrdU+/CD31+ ECs↑.
Zhu et al. (111)	Liraglutide	pMCAO	Permanent	Rat	–	1 h → 1 w, qd, s.c.	–	Sensory impairment↓, Aβ↓, NeuN↑, GFAP↓, Iba-1↓, TUNEL↓, Bcl-2↑, Bax↓.
Filchenko et al. (112)	Liraglutide	tMCAO	30	Rat	1 w, qd, s.c.	–	Diabetic	Infarct volume↓, neurological deficit↓.
He (113)	Liraglutide	pMCAO	Permanent	Mice	–	1 w, qd, s.c.	–	Neurological deficit↓, BDA-labeled axons↑, mitochondrial activities (ICDH↑, α-KG↑, DH↑, SDH↑), oxidative stress markers (cell viability, ATP levels↑, NeuN↑, LDH release↓, GAP-43↑, ROS↓, MMP↑, Fis1↓, complex mitochondrial-I↑).

**Table 2F:** A review of preclinical studies of GLP-1 and GLP-1RAs in stroke.

**References**	**Substance**	**Stroke model**	**Occlusion time (min)**	**Species**	**Timing of administration**		**Comorbidity**	**Main outcomes**
					**Pre-ischemia**	**Post-ischemia**		
Basalay et al. (114)	Liraglutide /	tMCAO	90/120/180	Rat	–	Onset, i.v.	–	Lir: Infarct volume↓, neurological deficit↓.
	Semaglutide				5 min before reperfusion, s.c.		–	Sema: Infarct volume↓, neurological deficit↓.
	Exendin 9-39				15 min before Sema, i.v.		–	Neuroprotection by Sema was abolished by Ex 9-39.
Shi et al. (115)	Liraglutide	pMCAO	Permanent	Rat	–	1 h, i.p.	Diabetic	Lir: Blood glucose↓, neurological deficit↓, infarct volume↓, oxidative stress (SOD↑, MPO↓), Kir6.2↑, SUR1↑.
	Insulin				–	1 h, i.p.		Ins: Blood glucose↓.
Li et al. (116)	Liraglutide	tMCAO	120	Mice	–	Onset → 2 d, q4 h	Diabetic	Infarct volume↓, PAWR↑, Haptoglobin (Hp)↓, Bcl-2↑, Bax↓, Serum amyloid A protein (SAA)↓, synapsis-related proteins↑ (Dpysl2, Syn1, Bsn, Map1b, Nf1, and Pde2a).
Abdel-latif et al. (117)	Lixisenatide	BCCAO	30	Rat	–	1 and 24 h, i.p	–	Lixi: Neurological deficit↓, GSH↑, catalase enzyme↑, MDA↓, caspase-3↓, TNF-α↓, VEGF↑, infarct volume↓, eNOS↑, exerted effects via GLP-1R dependent and independent pathways.
	Exendin 9-39				–	1 and 24 h, i.p.	–	Ex 9-39: Reversed some of the protective effects of Ex-4.

**Table 2G:** A review of preclinical studies of GLP-1 and GLP-1RAs in stroke.

**References**	**Substance**	**Stroke model**	**Occlusion time (min)**	**Species**	**Timing of administration**		**Comorbidity**	**Main outcomes**
					**Pre-ischemia**	**Post-ischemia**		
Abdel-latif et al. (118)	Lixisenatide/	BCCAO	30	Rat	2 w, qd, i.p.	–	Diabetic	Lixi: Insulin resistance↓, HOMA-IR↓, TG↓, LDL-C↓, NOX2↓, neurological deficits↓, oxidative stress markers (MDA↓, NOS↓, GSH↑), caspase-3↓, TNF-α↓, eNOS↓, infarct volume↓.
	Glimepiride				2 w, qd, p.o	–		Insulin resistance↓, HOMA-IR↓, TG↓, LDL-C↓, neurological deficits↓, oxidative stress markers (GSH↑), caspase-3↓, TNF-α↓, iNOS↓, NOX2↓, infarct volume↓.
Gad et al. (119)	Lixisenatide	BCCAO	60	Rat	2 w, qd, i.p.	–	–	Lixi: MAPK (p-P38↓and p-ERK↑), oxidative stress (MDA↓, SOD↑, GSH↑), TLR↓/NF-κB↓, IL-1β↓, MPO↓, TNF-α↓, Bax↓, Bcl-2↑, caspase-3↓.
Yang etal. (120)	Semaglutide	pMCAO	Permanent	Rat	–	2 h → 2 w, q2 d, i.p.	–	Neurological deficit↓, p38 MAPK↓, Iba-1↓, DCX↑, MKK↓, NF-κB p65↓, c-raf↑, c-Jun↓, Bcl-2/Bax↑, ERK2↑, caspase-3↓, ERK1/2↑, p-ERK1/2↑, IRS-1↑, p-IRS-1↑, nestin↑, CXCR4↑, SDF-1↑.
Zhang et al. (121)	DMB/	tMCAO	60	Mice	30 min, p.o.	–	–	DMB: Infarct volume↓, neurological deficit↓, cAMP↑/PKA↑/p-CREB↑, Bcl-2↑, Bax↓.
	Exendin-4/				30 min, i.p.	–	–	Ex-4: Infarct volume↓, neurological deficit↓.
	Exendin 9-39							Ex 9-39: blocked the beneficial effects of Ex-4 but not DMB.

**Table 2H:** A review of preclinical studies of GLP-1 and GLP-1RAs in stroke.

**References**	**Substance**	**Stroke model**	**Occlusion time (min)**	**Species**	**Timing of administration**		**Comorbidity**	**Main outcomes**
					**Pre-ischemia**	**Post-ischemia**		
Han et al. (125)	DA	tMCAO	60	Rat	–	1 h, i.p.	–	DA: Neurological deficit↓, infarct volume↓, Bcl-2↑, TUNEL↓, Bax↓, iNOS↓.
	Val(8)GLP-1-Glu-PAL				–	1 h, i.p.	–	Val(8): Infarct volume↓, neurological deficit↓, Bcl-2↑, TUNEL↓, Bax↓, iNOS↓.
Bai et al. (126)	DA3-CH	tMCAO	120	Rat	2 w, qd, i.p.	–	Diabetic	DA3: Neurological deficit↓, infarct volume↓, CHOP↓, NeuN↑, GRP78↓, Bax↓, Bcl-2↑, caspase-12↓.
	Liraglutide				2 w, qd, i.p.	–	–	Lir: Neurological deficit↓, infarct volume↓, Bcl-2↑, NeuN↑, GRP78↓, CHOP↓, Bax↓, caspase-12↓.
Li et al. (127)	OXM	tMCAO	60	Rat	15 min, i.c.v	–	–	CAMP↑, GLP-1R↑, p-CREB/CREB↑, PKA↑, MAPK, cell viability↑, infarct volume↓, neurological deficit↓.
Wang et al. (128)	P7C3	tMCAO	40	Mice	–	2 h → 3 d, qd, i.v.	–	P7C3: Survival rates↑, neurological deficits↓, infarct volume↓, BBB leakage↓, p65 NF-κB↓, iNOS↓, caspase-3↓, a-caspase-3↓, DCX↑, β-tub3, ki67, BrdU↑, adam11↑, adamts20↑, SpGSK-3↑, Bcl-2↑, p-PKA↑, p-Akt↑, p-catenin↑, cAMP↑, and dependent of GLP-1R.
	Exendin-4/ Exendin 9-39				–/ 15 min, i.v.	2 h → 3 d, qd, i.v./2 h, i.v.	–	Ex-4: Survival rates↑, cAMP↑. Ex 9-39: blocked protective effects of P7C3.

*↑, enhancement; ↓, reduction; i.p., intraperitoneal; s.c., subcutaneous; i.c.v., intracerebroventricular; i.n., intranasal; t.v., transvenous; p.o., oral; MCAO, middle cerebral artery occlusion; p/tMCAO, permanent/transient; BCCAO, bilateral common carotid artery occlusion; TUNEL, terminal deoxynucleotidyl transferase (TdT)-mediated dUTP-biotin nick-end labeling; ROS, reactive oxygen species; M2, anti-inflammatory macrophages: marked by CD206, arginase1 and YM1/2; VEGF, vascular endothelial growth factor; p-Akt, phosphorylated protein kinase B; ED1, a marker of activated microglia; 8-OHdG, 8-hydroxy deoxyguanosine; p-CREB, phosphorylated cyclic AMP (cAMP) response element binding protein; COX-2, cyclo-oxygenase-2; MDA, malondialdehyde; PKA, protein kinase A; DCX, doublecortin; NSE, neuron specific enolase; MCP-1, monocyte chemotactic protein 1; iNOS, inducible nitric oxide synthase; SOD, superoxide dismutase; Bax, Bcl-2-associated X protein; DNP, dinitrophenol; GSH, reduced glutathione; GFAP, glial fibrillary acidic protein; PI3K, phosphoinositide 3-kinase; γH2AX, Histone H2A (Lys119); NeuN, neuronal nuclei protein; APE1, apurinic/apyrimidinic endonuclease 1; CHOP, CCAAT/-enhancer-binding protein homologous protein; GSK3β, glycogen synthase kinase 3β; NF-κB, nuclear factor kappa-light-chain enhancer of activated B cells; IL-1β, Interleukin-1 beta; Bcl-2, B-cell lymphoma 2; HIF-1α, hypoxia-inducible factor 1-alpha; gp91, glycoprotein91; p-eNOS, phosphorylated endothelial nitric oxide synthases; ICAM-1, intercellular adhesion molecule 1; HHE, 4-hydroxy 2-hexenal; DHE, dihydroethidium; cAMP, cyclic adenosine monophosphate; PARP, poly ADP-ribose polymerase; BBB, blood brain barrier; 8-OHdG, 8-Oxo2'-deoxyguanosine; MPO, myeloperoxidase; HO-1, heme oxygenase-1; MBP, myelin basic protein; Nrf2, nuclear factor erythroid-2; Ki67, nuclear proliferation antigen; Iba-1, ionized calcium-binding adapter molecule-1; SDF-1, stromal cell derived factor-1*.

### GLP-1, RhGLP-1 (Recombinant Human GLP-1), and ProGLP-1 (Long Acting GLP-1RA)

Because GLP-1 receptors are expressed in the CNS and GLP-1 has also been revealed to play a protective role in cerebral ischemia, GLP-1 may be a promising drug for stroke. However, the short half-life and dose-limited adverse gastrointestinal effects of GLP-1 have limited its clinical application. Some forms of GLP-1, which are processed from proglucagon, differ in the potency to increase glucose-induced insulin secretion.

One study (87) provides evidence that pro-GLP-1 prevented apoptosis induced by oxygen-glucose deprivation (OGD) in cultured cortical neurons, and exerted neuroprotective effects through the cAMP/PKA and PI3K/Akt signaling pathways but not the ERK pathway in mice against cerebral ischemia. Since pro-GLP-1 made no difference to levels of insulin and blood glucose, and the neuroprotective effect was blocked when knocked down the GLP-1 receptor in the hippocampus, it can be inferred that pro-GLP-1 exerted neuroprotective effects against cerebral ischemia *via* activation of the GLP-1 receptor.

The recombinant human GLP-1(7-36) [RhGLP-1 (7-36)], a biosynthetic agent belonging to the GLP-1 RAs, is also applied to treat T2DM clinically. Without amidation in the C- terminal, RhGLP-1 (7-36) is the complete amino acid sequence of GLP-1 (7-36)-NH2 and is more homologous to human as compared to other GLP-1 RAs such as exenatide (122). However, RhGLP-1 (7-36) is a short-acting dosage form and its effects could be terminated more promptly when adverse events occur than long-acting GLP-1 RAs. Pretreatment with rhGLP-1 for 2 weeks can reduce blood glucose, body weight, and infarction volume, improve neurological deficits in diabetic rats after stroke compared to insulin (88). More concretely, RhGLP-1 lowered blood glucose in a dose-dependent manner while decreased the blood levels of biomarkers S100 Calcium Binding Protein B (S100B), major basic protein (MBP), and neuron specific enolase (NSE) which reflect cerebral damage (90) when administration implemented 2 weeks before transient middle cerebral artery occlusion (tMCAO) in diabetic rats. RhGLP-1 also decreased the level of cleaved caspase-3 and increased the expression of excitatory amino acid transporter 2 (EAAT2) and the ratio of Bcl-2/Bax from the level of protein (90). In another study (86), RhGLP-1 significantly increased the density of surviving neurons, alleviated oxidative stress parameters, and promoted vascular proliferation when pretreated with only a single dose before performing permanent middle cerebral artery occlusion (pMCAO) in rats with diabetes. In a separate study, Similar results were found by a second study (89) when rhGLP-1 was administrated from 2 h to 3 days after MCAO. The neuroprotective effect of rhGLP-1 might be based on promoting expression of the phase II detoxification enzyme HO-1, activating PI3K to up-regulate expression of antioxidant enzyme SOD, and nuclear transfer of Nrf2 proteins.

GLP-1(9-36) NH2 is formed by the DPP-4 enzyme cleaving the N-terminal dipeptide His-Ala of GLP-1(7-36) NH2 (123), which is a ligand for GLP-1 receptor with low affinity. Not much is known about the underlying mechanisms and efficacy of GLP-1 (9-36) in cerebral ischemia and reperfusion (CIR) injury (that is, middle cerebral artery was obstructed for some time and then achieved reperfusion). Studies have been shown that reperfusion has the potential to cause subsequent injury in ischemic tissue, which is called ischemia and reperfusion injury (124). It was reported that GLP-1 (9-36) decreased the level of nuclear factor kappa-B (NF-κB) in astrocytes after oxygen-glucose deprivation/reoxygenation (OGD/R) damage and inhibited neuronal apoptosis around the infarct area against CIR, the mechanism of which might be relying on the activation of the IGF-1 receptor rather than the GLP-1 receptor (91).

### Exenatide

Exenatide (Byetta® and Bydureon®, AstraZeneca), originally discovered as Exendin-4 (Ex-4) in the saliva of the gila monster (Heloderma suspectum), is a synthetic peptide with 39 amino acids (129). There is about 53% homology of amino acid sequence between the first 30 amino residues of Ex-4 and mammalian GLP-1, nevertheless, there is no similarity in the C-terminal non-apeptide extension. In contrast with GLP-1, there is a glycine at the second amino acid position in the N-terminus of Ex-4 that can protect the peptide against inactivation and degradation mediated by DPP-4 (130).

There has been evidence that intraperitoneal injection of Ex-4 protected against ischemia-induced neuronal apoptosis potentially by upregulating expression of the GLP-1R mainly in GABAergic interneurons or astrocytes in the hippocampal CA1 region of gerbils (77). In a separate study, Gong et al. (131) proved the neuroprotective efficacy of intracerebroventricular injection (i.c.v.) of Ex-4 exerted after transient ischemia/reperfusion in rats and first suggested that Ex-4 played a neuroprotective role probably by stimulating the GLP-1R/β-endorphin signal pathway in hippocampal microglia (76).

Another study (78) showed that Ex-4 improved neurological deficits after stroke in mice, and the neuroprotective effects of Ex-4 were achieved when administrated intravenously acutely at the onset of stroke or 1 h later, but were lost at 3 h. It must be pointed out that the dose of Ex-4 administrated in the study (~400 mg/kg) was much higher than a clinical dose of Ex-4 taken by patients with T2DM (0.1–0.2 mg/kg). Another group (93) showed that clinical dose of Ex-4 also reduced neuronal damage, arrested microglial infiltration, and increased stroke-induced neuroblast formation and proliferation of neural stem cell (NSCs) when administrated 4 weeks before and 2–4 weeks after inducing stroke in diabetic rats. In the latter study (94), Ex-4 was found to exert neuroprotective effects in both aged T2DM/obese and healthy mice by administrating a dose of 50 mg/kg Ex-4 from 1.5 or 3 h after MCAO. The difference between these outcomes and the experiment by (78) at 3 h possibly results from different methods of evaluation for stroke. Microglia/macrophages assume a diversity of phenotypes depending on the microenvironment, such as the classical pro-inflammatory M1 phenotype and the regulatory or anti-inflammatory M2 phenotype, respectively (132, 133). After stroke there is an early/acute polarization to the anti-inflammatory M2 phenotype, nevertheless, there is an increased transition to the pro-inflammatory M1 phenotype over time (134). Interestingly, the up-regulation of M2 markers demonstrates that Ex-4 promotes the polarization toward the anti-inflammatory M2 phenotype post MCAO in both aged T2DM/obese and normal mice, implying a new mechanism based on Ex-4- mediated neuroprotective efficacy (94, 135). From the clinical perspective, the advantage of this finding is that diabetics can receive a treatment based on activation of GLP-1R targeting their diabetes (i.e., antihyperglycemic), as well as improving the prognosis of stroke. Intranasal administration of Ex-4 at a dose of 0.5 μg/kg daily for 7 days before MCAO in mice, rather than intraperitoneal administration at an equivalent dose, exerted neuroprotective effect by reducing infarct volume and neurological deficits which were blocked by knocking down GLP-1 receptor with shRNA (97). Following activation of the GLP-1 receptor, the main signal pathway includes activation of adenylyl cyclase by stimulating Gα, which in turn stimulates the both the PI3K/Akt and cAMP/PKA/CREB pathways that regulate functions of various cells (136). The study indicates that intranasal administration (i.n.) may be a more effective mode of administration against cerebral ischemia as Ex-4 could be transported across the BBB by fast anterograde axonal transport. Their results also showed Ex-4 (50 μg/kg) had neuroprotective effects at 1.5 and 3 h after stroke. Therefore, the difference between this study and that of Darsalia et al. was potentially due to the different modes of administration.

Though GLP-1 RAs have been recognized to improve glucose tolerance and induce sustained secretion of insulin after discontinuing therapy, yet no study has been conducted to determine whether Ex-4 plays a continuing role in protecting against stroke after cessation of therapy and the potential mechanisms. Zhang et al. (102) demonstrated that pretreatment with Ex-4 for a week, induced tolerance to cerebral ischemia that lasted for at least 6 days in the mouse brain after MCAO and this neuroprotective state was related to upregulation of IGF-1R which was mediated by GLP-1R and, following by activation of the IGF1R-regulated the PI3K/AKT/mTOR/HIF-1 pathway by binding to IGF-1. Another study (95) found that Ex-4 downregulated the level of hypoxia-inducible factor-1α (HIF-1α) under hypoxic conditions *in vivo* (SH-SY5Y cells and primary cortical neurons) and ischemic state *in vivo* (transient ischemia model of gerbil) *in vitro*. These studies suggest that Ex-4 regulates the level of HIF-1α in the nerve cell which may play a significant part in the neuroprotection of Ex-4 against hypoxic-ischemic. Ex-4 exerted neuroprotective effects independent of endothelin receptor in a similar experiment (92). Furthermore, GLP-1 and its analog (Ex-4) enhanced DNA repair and protected cortical neurons in brains of rats after ischemia which were due to up-regulation of apurinic/apyrimidinic endonuclease 1 (APE1) mediated by PI3K/AKT/CREB pathway (100). APE1 is the most abundant apurinic endonuclease in human cells (137). Previous studies demonstrated that DNA repair efficiency of base excision repair can be improved by up-regulated APE1 (138, 139). In a similar study (103), chronic activation of GLP-1 receptors by Ex-4 promoted the recovery of forepaw grip strength correlated with counteracted atrophy of parvalbumin+ interneurons, normalized glycaemia, and insulin sensitivity as well as the pericyte coverage and density of microvessels, and restored formation of fibrotic scar at the stage of recovery in diabetic mice after stroke. The results proves that GLP-1 RAs are effective in trials of post-stroke rehabilitation in T2DM. However, the Ex-4-mediated rehabilitation was minor in non-diabetic mice. In effect, these mice make a quick recovery after stroke and, as a result, there is little chance that further improvement in recovery may be offered through pharmacological treatment.

Oxidative stress is one of the mechanisms underlying neuronal damage which may be caused by the acute brain ischemia (ABI) (140). Ex-4 offered protection against MCAO-induced disorder of the cerebral blood flow (CBF), expression of reactive oxygen species (ROS) in the blood and brain, production of oxidative stress-related and inflammatory proteins, dyskinesia and cognitive dysfunction, and contraction of the bladder in mouse with diabetes (99). In the study, they observed that Ex-4 treatment activated the Phospho- Akt/Phospho- endothelial nitric oxide synthase (p-Akt/p-eNOS) signaling pathway after MCAO, suggesting the recruitment of signal pathways which can protect against oxidative stress. It has been shown that synthetic biodegradable polyesters such as poly (D,L-lactide-co-glycolide) (PLGA) can be applied to increase the biological activity of administered drugs (141). In order to overcome the limited efficacy and short half-life of Ex-4, Chien et al. made use of a solvent-compatible microfluidic chip based on phenol formaldehyde resin to fabricate Ex-4- loaded PLGA (PEx-4) microspheres. Compared with Ex-4, PEx-4 showed sustained release of Ex-4 into the CSF and plasma over 2 weeks (96). Bilateral common carotid arteries occlusion (BCCAO) model is used to simulate the clinically transient global cerebral ischemia which is caused by cardiac arrest or severe hypotension (142). In comparison to Ex-4, PEx-4 was more efficient in improving BACCO-induced cognitive impairment and cortical edema in diabetic rats (96). Furthermore, the neuroprotective outcomes of PEx-4 were related to the suppression of GFAP-induced neurodegeneration, gp91/CHOP-regulated endoplasmic reticulum stress (ERS), NF-κB/ICAM-1-mediated inflammation, and Bcl-2/Bax/caspase-3/PARP-involved apoptosis. These effects are inspiring for the possibly therapeutic application of GLP-1 RAs in neurological and cardiovascular complications of diabetes.

As a major source of inflammatory cytokines in lesions after AIS, astrocytes play an important role in recruiting peripheral immune cells, activating microglia, aggravating brain injury, and destroying the BBB. Ex-4 relieved the leakage of the BBB while reduced levels of astrocyte-derived MCP-1, CXCL-1 MMP-9, and VEGF-A in ischemic areas after MCAO in rats (101). PI3K/Akt is the commonest downstream signal pathway activated by GLP-1 receptor and has been shown to be activated by Ex-4 in animal models of stroke. In addition, they found that Ex-4 influenced astrocytes subjected to OGD/R by down-regulating the protein of phosphor- Janus Kinase 2 (pJAK2)/signal transducer and activator of transcription 3 (STAT3), which is a novel and previously undiscovered GLP-1 signaling pathway (101). Based upon these results, disruption of the BBB and ischemia-induced inflammation could be ameliorated by Ex-4 in an astrocyte-dependent way.

Powerful internal mechanisms of protective efficacy among organs are activated *via* remote ischemic conditioning (RIC) which could be activated by cycles of ischaemia/reperfusion (I/R) exerted to a tissue or an organ which is far from the tissue/organ being sheltered (143). A large number of experiments indicated the protection of the RIC in brain and heart against I/R- induced damage. A different study (104) suggested that GLP-1R-mediated neuroprotective effects against ischemic stroke in rats might be established by the RIC, which were antagonized by highly selective GLP-1 receptor antagonist Ex 9-39. Moreover, GLP-1 receptors are expressed in the cells lining cortical arterioles, and Ex-4 efficiently reversed the constriction of the arterioles induced by OGD or lactate *in vitro* and increased the CBF *in vivo*. Consequently, GLP-1R-induced neuroprotection may be mediated by its effects on cortical arterioles as well as ameliorated perfusion of the peri-infarct areas in the brain.

Cardioembolic strokes, most frequently caused by atrial fibrillation (AF), are associated with worse outcomes, and a higher risk of hemorrhagic transformation (HT) compared with ischemic strokes from other causes (144–146). But so far, there seems to be no effective therapy to prevent HT in routine clinical practice. Chen et al. (71) observed that Ex-4 can restrain neuroinflammation and stabilize the BBB via PI3K/Akt-mediated suppression of glycogen synthase kinase-3β (GSK-3β) in the brain after warfarin-associated HT post-cerebral ischemia of mice. These findings might be of significant value clinically and would be particularly beneficial for patients who receive anticoagulant therapy. Considerable attention should be paid to the safety and efficacy of this therapy in future clinical trials.

### Liraglutide

Liraglutide (Victoza®, Novo Nordisk) is modified from human GLP-1 (hGLP-1 7–37) with approximately 97% homology to GLP-1, containing a C16 palmitoyl fatty-acid side-chain at Lys26 and a ser34Arg amino-acid substitution. Liraglutide is a stable GLP-1R agonist used clinically to treat T2DM with adverse effects similar to Ex-4 (147). A randomized, multinational study showed that liraglutide was superior to Ex-4 on glycemic control with good compliance (148). A single administration of liraglutide post- stroke decreased MCAO-induced infarction with behavioral improvement, and the neuroprotective effects of liraglutide in normoglycemic rats may be due to suppression of oxidative stress and upregulation of VEGF in the cerebral cortex, but not striatum (105). This study might have uncovered the neuroprotective potency of liraglutide against cerebral ischemia for the first time.

It has been determined that nuclear factor erythroid-2 (Nrf2)/heme oxygenase (HO-1) signal pathway plays a strong part in antioxidant stress and the overexpression of Nrf2 can dramatically reduce ischemic brain damage (149). Liraglutide activated the Nrf2/HO-1 pathway and protected cerebral neurons against stroke in diabetic rats (109). In another study (115), liraglutide significantly inhibited oxidative stress and inflammatory activation compared with insulin in diabetic rats after pMCAO, which is connected with the activation of mitoKATP channels. These outcomes are in agreement with previous experiments, suggesting that diabetes-aggravated ischemic damage was resulting from multifactorial interactions and normalization of hyperglycemia alone is not the major mechanism of neuroprotective effects. Further, it is confirmed that liraglutide is a neuroprotective drug that can directly protect against ischemic injury in animals with diabetes mellitus.

It has been shown that the GLP-1 RAs can reduce the mortality of cerebrovascular accidents in diabetics in clinical trials (150), but GLP-1 RAs are often used in combination with other agents, thus drug interactions cannot be ruled out. A different study (112) found that liraglutide markedly lessened cerebral infarction without causing hypoglycaemia in non-diabetic rats. Though both metformin and liraglutide contributed to euglycemia in experimental T2DM of rats, only liraglutide alleviated cerebral injury caused by stroke when compared with metformin. Liraglutide furthermore improved symptoms after stroke compared to insulin in rats with diabetes induced by streptozotocin (106). However, this diabetic model simulates type 1 diabetes mellitus (T1DM) and levels of blood glucose were not monitored in the course of treatment.

Neurons are the most oxygen-sensitive cells in the human body. It was shown that liraglutide clarify protection against hypoxia by activating the MAPK and PI3K/AKT pathways *in vivo* and *in vitro* against ischemic damage (107). What's more, liraglutide ameliorated motor and sensory disorders *via* inhibition of neuronal apoptosis *in vivo* (107). Focal cerebral infarction after distal MCAO not only causes the primary cortical infarction, but also leads to secondary injury in the regions that have synaptic connections with the primary ischemic lesion (151). The most common secondary damage is ipsilateral thalamic degeneration after cerebral infarction, including axonal degeneration, neuronal loss, gliosis, β-amyloid (Aβ) deposits, and hyperphosphorylation of Tau protein (152, 153). Such thalamic damage results in delayed neuronal recovery (154). A separate study (111) found liraglutide alleviated sensory deficit after focal cerebral ischemic stroke, which may be related to the improvement of Aβ3-16 deposition as well as secondary damage in the ipsilateral thalamus. This may provide a basis for finding new therapeutic targets for ischemic stroke. Nevertheless, whether liraglutide could reduce other types of Aβ requires more detailed elucidation. Studies have revealed that acute neuroprotection is provided by the administration of GLP-1 RAS prior to or immediately after inducing experimental stroke. However, drugs that provide long-term effects of treatment and/or target later phase after stroke might be more crucial, because a prolonged time-window of treatment applies to most of new victims, as well as dysfunction is persistent in patients with stroke (7, 155). Dong et al. (108) found delayed treatment with liraglutide from the first day to the fourth week after stroke could promote neurovascular remodeling and increase expression of GLP-1R, which helped to increase metabolism of glucose and improve neurological deficit in rats when initiated 24 h after tMCAO. By means of PET- imaging and other techniques, the study demonstrated a dose-dependent recovery of metabolism and function occurred after treatment with liraglutide. This may be the first study to show that delayed administration of liraglutide can treat cerebral ischemia. Cerebral repair after stroke calls for different processes, which contains synaptogenesis, neurogenesis, and angiogenesis (156). Neovascularization after ischemic injury in the brain improves microperfusion of tissue in the peri-infarct area (157). A separate study (110) confirmed that delayed therapy with liraglutide (24 h post-stroke and once daily for 2 weeks) can promote angiogenesis and long-term rehabilitation of cerebral ischemia in mice by up-regulating the expression of vascular endothelial growth factor (VEGF) in normoglycemic animals. It is acknowledged that axonal sprouting is a key factor for functional recovery of mice following a stroke (158). Liraglutide promoted axonal sprouting in primary cortical neurons exposed to H_2_O_2_ and in a pMCAO model in mice (159). In addition, the effect might be mediated by Sirt1-dependent mitochondrial improvement. These findings might be of clinically significance because chronic administration of liraglutide prior to and after stroke can improve stroke outcomes in diabetics while playing an antidiabetic role.

A recent proteomic study (116) explored the physiological protection of GLP-1 RAS during the progression of cerebral ischemia/reperfusion (CI/R) injury in mice. These proteomic data showed that liraglutide exerted a variety of effects on the phosphorylation and expression of proteins in MCAO mice. Specifically, liraglutide downregulated expression of Haptoglobin (Hp), upregulated levels of PRKC apoptosis WT1 regulator (PAWR), and synapse-related proteins including Syn1, Pde2a, Dpysl2, Nf1, Bsn, and Map1b, and increased the densities of neurons and synapses. The results of this study may help identify novel therapeutic targets for ischemia-reperfusion.

### Lixisenatide

Lixisenatide (Lyxumia®, Sanofi), an analog of Exenatide, is formed by omitting the proline at position 38 as well as adding six sequential lysine residues to the C-terminus (160). It is a GLP-1R agonist used to treat T2DM with neuroprotective properties. Furthermore, lixisenatide can cross the BBB at very low doses with obvious physiological activity, and promote neurogenesis in the CNS (58).

Along with T2DM-induced hyperglycemia, cerebral blood flow (CBF) is obviously decreased on account of regulation of vasculature and vasoactive mediators, especially endothelium-derived nitric oxide (NO) (161). It is important to note that a sustained reduction in NO in the CBF leads to poor prognosis in patients with ischemic stroke (162).

It was revealed that pretreatment with lixisenatide significantly suppressed elevation of inducible nitric oxide synthase (iNOS) and reversed the expression of endothelial nitric oxide synthase (eNOS) at the protein level and reduced mRNA of (NADPH oxidases 2) NOX2 in carotid arteries, which finally alleviated the endothelial dysfunction caused by ischemic/reperfusion (CI/R) in rats with diabetes more apparently than glimepiride (118). Glimepiride, a once-daily sulfonylurea antidiabetic drug, was selected as therapeutic comparator in this study, which acts through enhancing insulin secretion from β cells *via* different mechanisms (163). The data indicate that both glimepiride and lixisenatide dampened the parameters of vascular oxidative stress such as NOX2 and iNOS partly due to glycemic control. Nevertheless, the effects of lixisenatide on vascular improvements are better than that of glimepiride, which might be in connection with activation of GLP-1R. In another study (117), two doses of lixisenatide (1, 10 nmol/kg) were administered, respectively, to rats post- stroke. In both groups, lixisenatide markedly ameliorated neurological deficit, reduced infarct volume, along with inhibited the expression of oxidative stress parameters (GSH, MDA, NO and catalase enzyme), apoptotic marker (caspase-3), and inflammatory factor (TNF-α) in ischemic brains of rats (117). It's worth noting that these protective effects are independent of GLP-1R activity because they weren't blocked by Ex 9-39. However, the expression of VEGF protein increased by lixisenatide was inhibited by adding Ex 9-39. Moreover, the effects of the low dose by lixisenatide on stroke was superior to that of high dose. One probable interpretation is that transport of molecules across the BBB is highly controlled and the ingestion of the abnormally high doses of drug may affect this. Another explanation is that large doses of lixisenatide induce desensitization of GLP-1 signals in the brain. Nevertheless, further trials are needed to confirm the hypothesis. Moreover, one recent study (119) showed that lixisenatide exerted neuroprotection potentially by downregulating levels of NF-κB, TLR2/4, MPO, and pP38 as well as upregulating expression of pERK1/2. Thus, this study suggests that neuroprotective effects of lixisenatide may be related to the TLR/MAPK pathway following CI/R.

### Semaglutide

Semaglutide (Ozempic®, Novo Nordisk), a modification of liraglutide, has a 94% homology with human GLP-1. It becomes protease-resistant by changing the amino acid at position 8 and an extended spacer for the attached fatty acid which prolongs the half-time in the blood (164), and is a once-weekly GLP-1R agonist for T2DM. Oral GLP-1 RA is a modified version from its subcutaneously administered semaglutide (165). Semaglutide has shown good neuroprotective effects in animal models of PD, and a phase II clinical trial is currently ongoing (166, 167). The neuroprotective effects of limiting infarct and improving neurological function by semaglutide are dose-dependent against tMCAO in non-diabetic rats and are at least as strong as liraglutide (114). Further, the GLP-1R antagonist Ex 9-39 eliminated the neuroprotective effects of semaglutide in this study, implying that GLP-1Rs play a key role in these effects. These results are in accord with the prior work by Darsalia et al., where Ex-4 exerted neuroprotective efficacy in wild type rather than GLP-1R^−/−^ mice. Recently, we found that semaglutide therapy post-stroke can prevent ischemia-induced neurotoxicity and normalize neurogenesis and proliferation of stem cells in the brain *via* inhibition of p38 MAPK/MKK/c-Jun activity, regulation of Bcl-2/Bax pathways, and C-raf/ERK/caspase-3, restoration of insulin signaling sensitivity, and normalization of the ERK1 and IRS1 pathways (120). What's more, we continuously measured blood glucose in rats which were administrated semaglutide from 2 h to day 14 following pMCAO, further proved the neuroprotective effects without causing a hypoglycemic episode.

### DMB (GLP-1Ragonist/Modulator)

The GLP-1R agonist quinoxaline 6,7-dichloro-2-methylsulfonyl-3-N-tert-butylaminoquinoxaline (DMB; also known as Compound 2) is a unique small-molecule agonist based on quinoxaline and allosteric modulator of GLP-1Rs with the potential to add the affinity for its receptor, first discovered by Knudsen et al. (168).

Zhang et al. (121) found that pretreatment with DMB significantly reduced the neurological deficits and cerebral infarction caused by MCAO in mice. The neuroprotective effects of DMB were regulated by activating GLP-1 receptors and then stimulating the cAMP/PKA/CREB signaling pathway. However, it is reported that DMB interacts with the GLP-1 receptor at an allosteric site, which is thought to be a cavity located near the transmembrane 5 and 6 region (169). However, Ex 9–39 blocks action of GLP-1 by totally binding to the extracellular domain of GLP-1 receptor (170, 171). In this study, similar results were obtained where the neuroprotection of DMB was inhibited by the knockdown of GLP-1 receptor with shRNA but not by a GLP-1 receptor antagonist, and the cAMP induced by DMB was not suppressed by Ex 9-39 (121). In conclusion, these findings manifest that DMB has great potential in the treatment of cerebral ischemic stroke.

### Dual GLP-1/Glucose-Dependent Insulinotropic Peptide (GIP) Receptor Agonists (Dual GLP-1/GIPR Agonists, DA)

Currently, novel dual GLP-1/GIP receptor agonists have been developed (55), which are derived from an intermixed sequence of GLP-1 and GIP and have proved properties enhancing efficacy of insulinotropic and antihyperglycemic compared with selective GLP-1 receptor agonists such as liraglutide. The pharmacokinetic enhancement attenuated the peak of drug exposure combining with less dependence on GLP-1–mediated pharmacology, and avoided the gastrointestinal adverse reactions of selective GLP-1 receptor agonist. In other studies, it has been shown that dual GLP-1/GIPR agonists exert neuroprotective effects in animal models of AD and PD (83, 172).

One study (125) compared a novel dual GLP-1/GIP analog (DA-JC1) with Val(8)GLP-1 (glu-PAL) in rats experienced MCAO. Val(8)GLP-1 (glu-PAL), a modified version of liraglutide, has an enhanced biological half-life and has shown neuroprotective effects (173, 174). DA-JC1 was more effective against neuronal degeneration than Val(8)-GLP-1, with higher scores of neurological function and level of Bcl-2, but lower cerebral infarction, expression of iNOS and Bax, and percent of TUNEL-positive neurons in the group treated with DA. In brief, dual GLP-1/GIP receptor agonist might be more protective in crucial biomarkers of neurodegeneration in brain than a GLP-1– based agonist.

In another recent publication (126), it was shown that pretreatment with the dual-GLP-1/GIP receptor agonist DA3-CH or liraglutide could decrease the levels of ERS damage proteins (CHOP, GRP78,) and pro-apoptotic factors (Bax, Caspase12) as well as increase anti-apoptotic factor (Bcl-2), and reduce neuronal death and neurological damage after stroke in diabetic rats. What's more, the neuroprotective efficacy of DA3-CH is higher than that of liraglutide, a single GLP-1R agonist.

### Oxyntomodulin (Co-activates GLP-1R and Glucagon Receptor)

Oxyntomodulin (OXM), an endogenous proglucagon-derived intestinal peptide that co-activates the GLP-1 receptor and the glucagon receptor (GCGR), was isolated from porcine jejunoileum extract in 1981 (175). It was produced primarily in enteroendocrine L-cells and released together with GLP-1 in response to food intake. What's more, OXM can pass through the BBB *via* a mechanism similar to GLP-1 (176). Natural OXM peptide has both neuroprotective and neurotrophic effects against oxidative stress and glutamate toxicity in cultured primary cortical neurons of rat and SH-SY5Y cells, two cellular models that are broad applied to evaluating neuroprotective and neurotrophic effects of experimental therapeutics in the development of neuroprotective agents (127). Intracerebroventricular administration of OXM apparently decreased cerebral infarction and enhanced spontaneous activities after MCAO in rats. It seems that the effects primarily mediated by GLP-1R and subsequently via the PKA/MAPK signaling pathway.

### P7C3 (Aminopropyl Carbazole Compound)

A novel small molecule P7C3 with neuroprotective properties was discovered by means of a target-agnostic screening in living mice. It is an aminopropyl carbazole compound and orally bioavailable, can cross the BBB, and is atoxic when the dose is several times higher than the effective dose (177). Modified forms, including P7C3-A20 and P7C3-S243, have shown neuroprotection in rodent models of ALS, PD, traumatic brain injury (TBI), and age-related cognitive disorder (177). Lately, GSK-3 has been identified as an alternative target for drug-mediated neuroprotective effects against excitotoxicity of ischemic stroke in animal models because GSK-3 is a multifaceted protein with a variety of neurophysiological and cellular effects. GSK-3 inhibition has aroused widespread interest in the fields of neurogenesis, neurotrophy, neuroprotection, mood stabilization, and anti-inflammation (178). It's worth noting that the effects of P7C3 decreased the severity of brain damage (inflammation and apoptosis) and were closely associated with GSK-3 inhibition (128). P7C3 has also been proven to be an effective agent in promoting neurogenesis (179); nevertheless, the mechanism by which it works has yet to be elucidated.

Further, wang et al. (128) assessed the neurogenesis-associated effect of P7C3 post-stroke and clarified the molecular signaling mechanisms by using an experimental CI/R model in mice. To be specific, P7C3 increased the expression of neurogenesis- promoting and neuroprotective proteins, such as Bcl-2, doublecortin (DCX), adamts20, Ki67, adam11, and beta tubulin III (β-tub3), in the injured cortex and peri-infarct area. These protective effects of P7C3 may result from GSK-3 inhibition and enhanced expression of β-catenin, possibly through stimulation of the PI3K/Akt and cAMP/PKA pathways which are mediate by activation of GLP-1 receptor. The protective effects of P7C3 were blocked by Ex 9-39, but the use of Ex-4 improved the survival rate in comparison to that of P7C3. This might be the first report to illuminate that P7C3 promote neurogenesis mediated by allosteric activation of GLP-1 receptor and subsequently regulate levels of GSK-3/β-catenin after CI/R injury in mice.

## Discussion

During the past few years, there's increasing evidence from animal experiments that GLP-1 and GLP-1RAs are neuroprotective in stroke. These findings are quite reliable because they have already been replicated in a few laboratories and by using some animal models of stroke, with or without diabetes or hyperglycemia. We report a review focusing on preclinical trials to support GLP-1 RAs -targeted neuroprotective properties for ischemic-reperfusion damage in animal models of AIS.

**Figure 1:**
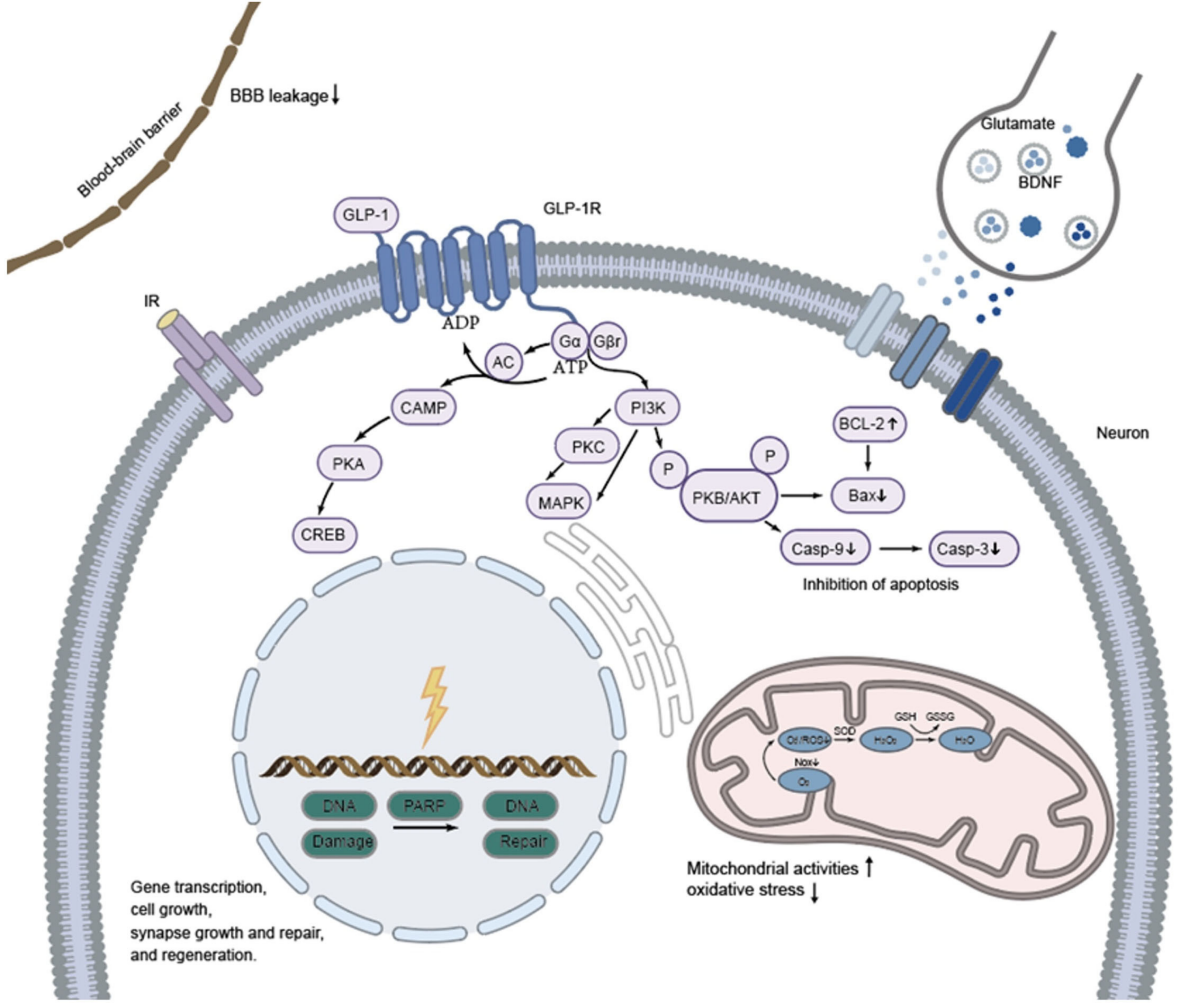
Proposed mechanisms of neuroprotective efficacy exerted by GLP-1 and GLP-1RAs against stroke in animals. Effects of GLP-1 and GLP-1RAs are mediated by binding to a specific, seven-transmembrane GLP-1R which is positively coupled to the adenylyl cyclase (AC) system. GLP-1 and GLP-1RAs acts directly by the cAMP/PKA signal pathway to facilitate gene transcription, synapse growth and repair, cell growth, and regeneration. The Gβγ dimer stimulates the PI3K, which then activates PKB/AKT pathway to inhibit apoptosis. In addition, GLP-1 and GLP-1 RAs play a role through reduction of blood-brain barrier leakage and neurotransmitter transmission among synapses as well.

It could be observed that ischemia in stroke models causes damage to multiple cells in the brain and blood vessels including neurons, glial cells (astrocytes and microglial cells), and vascular endothelial cells. In addition, increased permeability of both capillaries and the BBB is observed in the ischemic core, the penumbra, and other areas, resulting in perivascular and perineuronal edema. Activation of the GLP-1 receptor in microglia, GABAergic interneurons and astrocytes stimulates several intracellular pathways including the PI3K/Akt, cAMP/PKA/CREB, and P-Akt/p-eNOS and subsequently confers neuroprotective effects through inhibition of gp91/CHOP, Nrf2/HO-1, Bax/Bcl-2/caspase3/PARP, NF-κB/ICAM-1, and JAK2/STAT3. Schematic representation of the cell signaling pathways that are activated by GLP-1R stimulation and that exert the neuroprotection against insults that modeled stroke (see Figure 1). Once bound to GLP-1 receptor (a 7-transmembrane protein that belongs to the class B1 G-protein-coupled receptor family) (180), GLP-1 RAs (as GLP-1) initiate a signaling cascade that activates adenylyl cyclase (AC), increasing cAMP levels (in a dose-dependent manner) which, in turn, interacts with several downstream molecules, such as mitogen-activated protein kinase (MAPK), protein kinase A (PKA) and PI3K (181, 182). As an important downstream target of GLP-1 receptor activation, the cAMP/PKA pathway plays a key role in facilitating gene transcription, synapse growth and repair, cell growth, and regeneration. The Gβγ dimer stimulates the PI3K, which then activates PKB/AKT pathway to inhibit apoptosis. In addition, GLP-1 and GLP-1 RAs play a role through reduction of blood-brain barrier leakage and regulating neurotransmitter transmission (i.e., glutamate and BDNF) among synapses as well (183, 184). The neuroprotective effects are shown as reduced Infarct volume, improved motor and sensory impairments and cognitive function through inhibition of apoptosis, inflammation, and oxidative stress, reduced neuronal death and edema, stabilization of the BBB and promotion of the normalization of neurogenesis. Darsalia et al. hypothesized that neuroprotective effects of Ex-4 may occur through either GLP-1R-dependent or -independent pathways, in a dose-dependent manner (93). In addition, it has been revealed that pretreatment with lixisenatide suppressed expression of oxidative stress parameters (NO, GSH, MDA and catalase enzyme), apoptotic marker (caspase-3) and inflammatory marker (TNF-α) in ischemic rat brains independent on GLP-1R mediation. This means that GLP-1 RAs may exert effects in other ways as yet unknown. It should be noted that the timing (before, during or after stroke induction), mode (i.v., i.c.v., i.p.), and dose (low and high doses) of drug administration to different animals (gerbil, mice, rat) might exert different effects against stroke as we have described it above. Moreover, two main models including BACCO and MCAO (unilateral, bilateral) were used to mimic the ischemic state, which might also affect the results. The efficacy of receptor agonists is also different in animals with and without diabetes as levels of insulin and blood glucose can affect the pathophysiology and outcomes of stroke (185, 186). As GLP-1 RAs show neuroprotective effect such as reduce infarct volume and neurological deficits in normoglycemic model against stroke without affecting blood glucose levels, they may be potential candidates for the treatment of stroke alone. Indeed, reducing blood glucose level and insulin treatment did not improve neurological dysfunction. Moreover, the modified GLP-1 RAs, which are designed to activate the GLP-1 receptor more effectively, have stronger neuroprotective efficacy than GLP-1 RAs used in clinical practice.

There are some limitations in this review, as we did not include clinical trials or pay close attention to potentially protective mechanisms of agents in the pathophysiology of animal stroke. In line with the details reviewed by Marlet (187) and Erbil (188), we also found that there were very limited studies reporting negative results for neuroprotective effects of GLP-1 and GLP-1 RAs and publication bias favoring positive outcomes. Neuroprotection in the laboratory is studied primarily in rodent models of transient brain ischaemia but not in primate models. The review also included studies of bilateral stroke models that do not simulate naturally occurring strokes. Furthermore, we have incorporated one study of induced cerebral haemorrhagic transformation as well, as this event is also common in clinical practice.

Though many mechanisms have been presented, the definite mechanisms by which GLP-1 and GLP-1 RAs play a protective role have not been fully elucidated. The crucial question, of course, is whether these inspiring outcomes in preclinical trials can also translate into therapeutic effects in humans clinically. On the basis of the promising outcomes received in animal experiments, the potential translation of these agents for the therapy of stroke in clinical practice is highly possible. GLP-1 and GLP-1 receptor agonists may represent novel drugs of therapeutic intervention with potential value, providing an alternative to the existing therapy for stroke. It is, however, necessary to continue further investigations to test the neuroprotective mechanisms of GLP-1 and GLP-1 RAs. In the future, large-scale clinical trials are the necessary procedure to verify the results revealed in animal experiments and to guarantee their clinical application to patients suffering stroke. The indications, safety, efficacy, and mechanisms of action of GLP-1R agonists in AIS patients will be the focus of clinical trials.

## Author Contributions

All authors made substantial contributions to the work and approved the final version of the manuscript.

## Funding

This study received funding from Shanghai Municipal Key Clinical Specialty (shslczdzk02801), and the Shanghai Municipal Health Commission (grant number 2020YJZX0109). Received project funding from the Surface Nature Foundation and the project category is Natural Science Fund. The project title is the study of the neuroprotective mechanism of incretin therapy based on Wnt/b-catenin signaling pathway in Parkinson's disease. The project number is 201901D111383.

## Conflict of Interest

The authors declare that the research was conducted in the absence of any commercial or financial relationships that could be construed as a potential conflict of interest.

## Publisher's Note

All claims expressed in this article are solely those of the authors and do not necessarily represent those of their affiliated organizations, or those of the publisher, the editors and the reviewers. Any product that may be evaluated in this article, or claim that may be made by its manufacturer, is not guaranteed or endorsed by the publisher.
